# Electrochemical Microstructuring of Columnar Cu_2_O Layers Through Preferential Grain Boundary Dissolution

**DOI:** 10.3390/mi17080979

**Published:** 2026-08-19

**Authors:** Pei Loon Khoo, Mizuki Kono, Katsutoshi Sakai, Masakazu Kobayashi, Masanobu Izaki

**Affiliations:** 1 Graduate School of Engineering, Toyohashi University of Technology, Toyohashi 441-8580, Aichi, Japan; 2 Social Cooperation Center, Nara Women’s University, Nara-shi 630-8506, Nara, Japan; 3 Faculty of Electrical Engineering, National Institute of Technology, Nara College, Yamato-Koriyama-shi 639-1058, Nara, Japan

**Keywords:** Cu_2_O, electrodeposition, electrochemical microstructuring, intercolumnar opening, surface topography, columnar coating, oxidative material removal, photoelectrochemistry

## Abstract

Crystalline oxide microfeatures offer optical, electronic, catalytic, and interfacial functions, but their fabrication often requires templates, patterned scaffolds, or serial machining. A template-free route converted an electrodeposited Cu_2_O coating on Au(111)/Si into substrate-supported vertical microfeatures by anodization at a nominal cell voltage of 10 V in 0.002 mol L^−1^ Na_2_S_2_O_8_ at 277 K. FE-SEM showed progressive widening of the pre-existing intercolumnar network and narrowing of the retained features. This spatially non-uniform removal identifies preferential dissolution along the intercolumnar network as the principal removal pathway at the coating-morphology scale. From 1 to 8 min, the within-image mean and median feature widths decreased by 36.0% and 45.9%, respectively. The dominant out-of-plane Cu_2_O(111) diffraction signature was retained while mean visible reflectance decreased. The Cu-H_2_O potential-pH framework provides a qualitative thermodynamic guide to possible oxidative pathways that are evaluated against the experimental evidence. C 1s-referenced XPS provides direct ex situ evidence of an anodization-associated Cu(II)/CuO-like contribution at the outermost surface. Chopped photoelectrochemical measurements showed the largest condition-level light–dark current density contrast after short anodization, providing a secondary functional comparison of the completed coatings.

## 1. Introduction

Crystalline oxide coatings are widely used in optical, electronic, catalytic, and interfacial technologies, where microstructure strongly influences transport, surface accessibility, and functional response. However, producing well-defined vertical microstructures within these coatings commonly requires sacrificial templates, pre-patterned scaffolds, or serial top-down machining. Solution-based electrodeposition provides a scalable, lithography-free means of forming crystalline oxide coatings over areas determined primarily by the dimensions of the electrochemical cell. Accordingly, most electrodeposition research has focused on controlling oxide morphology during nucleation and growth through electrolyte composition, applied potential, temperature, substrate selection, and deposition templates [1,2,3].

Cuprous oxide (Cu_2_O) is a versatile p-type semiconductor with a reported band-gap range of 1.9–2.1 eV and strong visible-light absorption [4,5,6]. A high external quantum efficiency was reported at a wavelength near 415 nm for an annealed Cu_2_O/ZnO photovoltaic device. Together with an optical depth below 150 nm at wavelengths below 460 nm, this result indicates efficient collection of carriers generated close to the heterointerface [4,7]. This photovoltaic result highlights a broader principle for light-absorbing semiconductors: photogenerated carriers must reach a collecting or reactive interface before recombination. Vertically oriented, laterally separated features can retain axial thickness for light absorption while exposing sidewall interfaces and shortening radial carrier-transport distances. These considerations have sustained interest in Cu_2_O as a photovoltaic absorber and photocathode coating [1,4,6,8]. Electrodeposition is widely used to create oxide layers, whereas post-deposition anodic restructuring remains little explored. In this approach, a completed coating is restructured into microfeatures by anodically opening its pre-existing intercolumnar network. Vertical Cu_2_O architectures have been fabricated by template-assisted electrodeposition in porous alumina [9]. Aligned Cu_2_O-containing structures have also been prepared by multilayer shell formation [8]. Both approaches define the vertical geometry during templating or growth rather than by restructuring a completed coating.

Post-deposition dissolution offers a direct route for restructuring electrochemically formed oxides after growth. Niu et al. correlated electron backscatter diffraction with scanning electron microscopy after ammonia etching of thermally oxidized Cu_2_O and found orientation-dependent morphology, with the high-index crystal group etched more effectively than the low-index group [10]. Deuermeier et al. showed that polycrystalline Cu_2_O can contain chemically and electrically distinct grain boundary regions, including Cu(II) segregation associated with locally CuO-like conductivity [11]. In the present study, post-deposition anodic restructuring of an electrodeposited columnar Cu_2_O coating was examined. Oxidative material removal was expected to be promoted by anodic polarization under gas-evolving conditions, while preferential opening was expected to be guided by the coating’s intrinsic intercolumnar network. Throughout this work, the title term “preferential grain boundary dissolution” denotes the morphology-scale pattern of spatially non-uniform removal expressed as intercolumnar opening. The individual boundary regions were not crystallographically resolved, so the evidence-supported finding is preferential dissolution along the coating’s intercolumnar network.

A dense columnar Cu_2_O coating was first electrodeposited on Au(111)/Si and subsequently microstructured by high-potential, oxygen-evolving anodization in dilute, slightly acidic sodium peroxydisulfate. The morphology hypothesis predicts broader intercolumnar gaps, reduced lateral width of the remaining features, and greater sidewall exposure with increasing anodization time, while a substrate-supported vertical framework remains. FE-SEM and image analysis provide the primary test, while anodic charge density provides a complementary operational record of the gas-evolving anodization. XRD, reflectance, and surface-sensitive XPS provide complementary structural, optical, and chemical evidence, while chopped photoelectrochemistry provides a secondary post-anodization functional comparison. The demonstrated contribution is the conversion of a completed dense Cu_2_O coating into separated vertical microfeatures without sacrificial templates, lithographic patterning, or serial feature-by-feature machining.

## 2. Materials and Methods

### 2.1. Electrodeposition of Cu_2_O Layer

Cu_2_O coatings were deposited in a three-electrode cell using an Au(111)/Si substrate (Kobelco Research Institute, Inc., Kobe, Japan) as the working electrode, a Pt plate as the counter electrode, and an Ag/AgCl reference electrode (HS-205C, DKK-TOA Corporation, Tokyo, Japan, procured through Kirk Co., Ltd., Nagoya, Japan) containing saturated KCl (+0.198 V vs. NHE). Before deposition, the substrates were ultrasonically cleaned in acetone for 3 min and in deionized water purified using an Elix-UV system (Nihon Millipore K.K., Tokyo, Japan) for 1 min, followed by UV/O_3_ cleaning for 10 min (ASM 401OZ, Asumi Giken, Limited, Tokyo, Japan). The deposition area was defined as 10 mm × 10 mm with fluoropolymer glass-adhesive Nitoflon masking tape (Nitto Denko Corporation, Osaka, Japan). The aqueous bath contained 0.4 mol L^−1^ copper(II) acetate monohydrate (Nacalai Tesque, Inc., Kyoto, Japan, >99%) and 3.0 mol L^−1^ lactic acid (Kanto Chemical Co., Inc., Tokyo, Japan, 85.0–92.0%). The pH was adjusted to 12.5 using NaOH (Kanto Chemical, >97%) at room temperature. Deposition was performed without agitation at −0.4 V vs. Ag/AgCl until −3.0 C cm^−2^ was reached, while the bath was maintained at 313 K. A computer-controlled potentiostat/galvanostat (VERSASTAT 4, Princeton Applied Research, AMETEK Scientific Instruments, Oak Ridge, TN, USA) was used.

### 2.2. Electrochemical Microstructuring

After deposition, the Cu_2_O/Au specimens were mounted as vertically immersed planar working electrodes with a Pt counter electrode and an Ag/AgCl reference channel used to record the working electrode potential. Epoxy masked the coating edges, rear surface, and electrical contact, leaving an anodization area of 1.00 cm^2^ exposed. The electrolyte was 0.002 mol L^−1^ sodium peroxydisulfate (Na_2_S_2_O_8_, Kanto Chemical, >98%), with a measured pH around 6, and was maintained at 277 K with a double-walled jacketed electrochemical cell connected to a low-temperature thermostatic water circulator (LTB-250, AS ONE Corporation, Osaka, Japan). A Kikusui PWR2001H direct-current power supply (Kikusui Electronics Corporation, Yokohama, Japan) and Keithley 2461 high-current source meter (Keithley Instruments, LLC, Cleveland, OH, USA) were used for anodization and potential measurement. As shown in Table 1, nominal cell voltages of 5, 10, or 15 V were applied across the Cu_2_O-Pt cell, while the Cu_2_O working-electrode potential was recorded separately against Ag/AgCl and expressed vs. NHE. For the voltage screen, anodization was terminated after 10, 3, and 2 min, respectively, when reflective Au became visible near the coating edge. A nominal cell voltage of 10 V was selected for the 1–8 min series used to investigate the effect of anodization time. Each anodization duration was applied to a separately prepared Cu_2_O-coated specimen under the same nominal processing conditions. Anodization time was the only intentionally varied parameter. Current density and working electrode potential were also recorded during anodization.

### 2.3. Structural, Morphological, Surface, Optical and Photoelectrochemical Characterization

Out-of-plane θ–2θ X-ray diffraction (XRD) scans and pole figures were measured with a Rigaku SmartLab diffractometer (Rigaku Corporation, Akishima, Japan) using Cu Kα radiation at 40 kV and 50 mA. The θ–2θ profiles were recorded from 20° to 80° at 4° min^−1^. Separate fixed-2θ pole measurements were obtained for the Cu_2_O(220) reflection of the Cu_2_O/Au coating and the Au(220) reflection of the Au reference. Both showed an annular intensity maximum near ψ = 36°. Each background-corrected map is normalized to its own maximum, so color is comparable within a map but not as absolute intensity between maps. The stereographic maps were measured to ψ = 75°. No interpolation, smoothing, defocusing, absorption, or polarization correction was applied.

Surface and cross-sectional morphology was observed by field-emission scanning electron microscopy (FE-SEM, Hitachi SU8000 Type II, Hitachi High-Technologies Corporation, Tokyo, Japan) at 5.0 kV using secondary-electron upper-detector mode. The emission current was initially set to 10 µA and decreased during image acquisition. Cross-sectional specimens were prepared by mechanically scoring and splitting the supported samples. Top-view feature widths were measured with ImageJ (version 1.54f) at 400 locations for each anodization condition. The mean represents the average measured width, whereas the median represents the typical width and is less sensitive to unusually large values. The standard deviation describes absolute dispersion, and the coefficient of variation (standard deviation divided by the mean) describes dispersion relative to the mean. The scale recorded in each FE-SEM image was used to calibrate ImageJ before width analysis, and all 400 measurements per condition were obtained from the corresponding calibrated image and evaluated consistently using the same method.

Reflectance was measured from 350 to 1000 nm with a Hitachi U-4100 UV-vis-NIR spectrophotometer (Hitachi High-Technologies Corporation, Tokyo, Japan) using an integrating sphere and an Au/Si substrate as the baseline. For condition-level comparison, the arithmetic mean over 400–800 nm was used as a common broadband interval spanning the principal visible response and the Cu_2_O absorption-edge region.

Photoelectrochemical (PEC) measurements were conducted in a windowed H-cell using the Cu_2_O coating as the working electrode, a Pt counter electrode, and a reversible hydrogen electrode (RHE). The exposed area was 0.5 cm^2^. The electrolyte contained 0.5 mol L^−1^ sodium sulfate decahydrate (Kanto Chemical) and 0.1 mol L^−1^ sodium tetraborate decahydrate (Kanto Chemical). KOH (Kanto Chemical, 86.0%) adjusted the pH to 13.8, and the electrolyte was purged with Ar for 30 min. pH 13.8 was selected following the alkaline Cu_2_O/CuO photocathode procedure reported by Yang et al. [12]. PEC was used to evaluate coating function after anodization rather than reproduce the near-pH 6 anodization environment. Using the same condition enabled direct comparison of the planar and anodized photoresponses. For comparison within the present coating series, the chopped-light response was expressed as the absolute difference between the illuminated and dark current density branches near 0.20 V vs. RHE.

Linear sweep voltammetry (LSV) was performed using a VersaSTAT 4 potentiostat/galvanostat (Princeton Applied Research, AMETEK). The potential was swept from the open-circuit potential to 0 V vs. RHE at 5 mV s^−1^ under AM1.5G illumination chopped every 5 s. The exposed area was 0.5 cm^2^.

The Faradaic upper-bound calculation, its literature basis, and Equations (S1) and (S2) are reported in the Supporting Information. Because oxygen evolution and other anodic processes contribute to the current, the result represents the maximum removal scale compatible with the recorded charge rather than a direct measurement of dissolution efficiency, mass loss, removed thickness, or dissolved-copper concentration. Nonetheless, it provides a quantitative ceiling for comparing the charge record with the observed morphology evolution.

X-ray photoelectron spectroscopy (XPS) was performed on the as-deposited and the voltage-screen endpoint Cu_2_O subjected to anodization at 5 V for 10 min, 10 V for 3 min, and 15 V for 2 min. A PHI Quantera SXM-CI (ULVAC-PHI, Inc., Chigasaki, Japan) with monochromatic Al Kα radiation (1486.6 eV) was operated at 25.09 W with a 100 µm beam. Cu 2p_3/2_ and Cu LMM regions were acquired at a pass energy of 112 eV at the as-received surface and after Ar^+^ sputtering. Each region was independently corrected using adventitious C 1s position referenced to 284.8 eV.

## 3. Results and Discussion

### 3.1. Columnar Cu_2_O Electrodeposition Before Anodization

**Figure 1:**
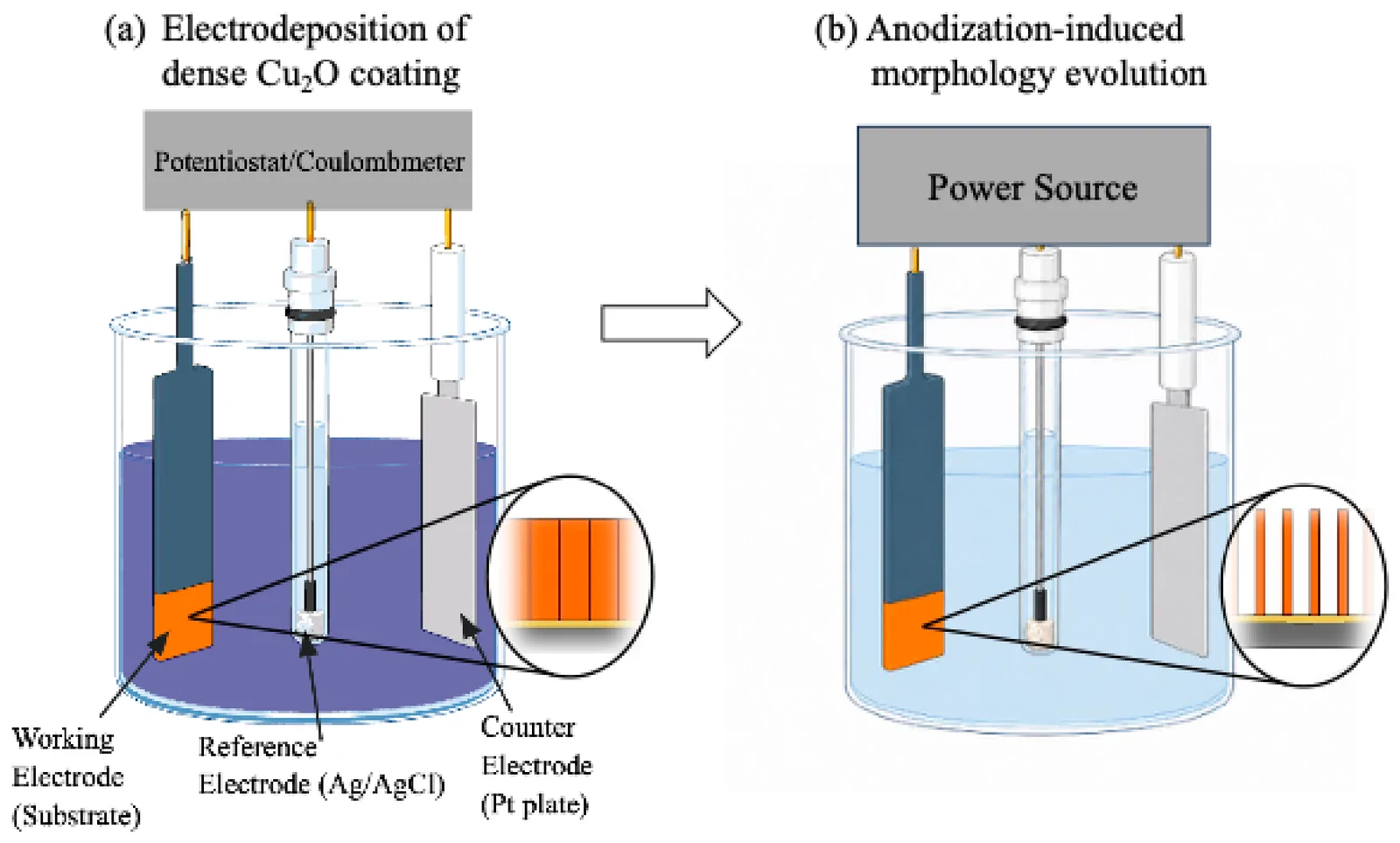
Two-step electrochemical route for microstructuring a columnar Cu_2_O coating. (**a**) Electrodeposition produces a dense columnar Cu_2_O coating with a thickness near 4 µm on Au(111)/Si. (**b**) The coated electrode was transferred to a separate 0.002 mol L^−1^ Na_2_S_2_O_8_ bath at 277 K and anodized at a nominal cell voltage of 10 V for 1–8 min under oxygen-evolving conditions.

Electrodeposition yielded a continuous Cu_2_O layer on Au(111)/Si. Figure 1 summarizes the two-step experimental route. Figure 1a shows electrodeposition of the dense coating of closely packed columnar Cu_2_O, and Figure 1b shows transfer of the completed coating to a separate peroxydisulfate bath for oxygen-evolving anodization. The cross-section in Figure 1a shows the vertically extended coating, whereas the corresponding surface is dense and lacks the open paths that emerge after anodization. The separated morphology was therefore created by restructuring a completed columnar coating rather than by directly growing isolated one-dimensional structures.

The starting diffraction pattern shows Cu_2_O(111) and Au(111) reflections near 36.6° and 38.2°, respectively. Electrodeposition of oriented Cu_2_O on thin Au/Si substrates has previously been demonstrated [2]. In the present data, the θ–2θ scan establishes strong out-of-plane preferred orientation. It is compatible with a (111) fiber texture in which neighboring columns share an approximately ⟨111⟩ surface normal but retain azimuthal rotation and a finite orientation spread.

### 3.2. Operational Selection of the Electrochemical Microstructuring Window

At nominal cell voltages of 5, 10, and 15 V, the current density records differed strongly and the voltage screen endpoints were 10, 3, and 2 min, respectively (Figure S1). Paired surface and cross-sectional FE-SEM images at these endpoints show that intercolumnar opening recurred across independently prepared coatings throughout the investigated voltage screen window. A nominal cell voltage of 10 V provided a practical interval from early intercolumnar opening to substantial material loss and was selected for the 1–8 min series (Figure S2). The separately recorded working electrode reading rose rapidly and approached a condition-dependent quasi-steady value. Vigorous microbubble evolution confirmed gas-evolving, non-equilibrium anodization. The working electrode potential includes uncompensated resistance, and the current was recorded in the presence of gas coverage at the electrode [13]. These measurements therefore describe the instrument-level anodization conditions rather than corrected interfacial potentials or equilibrium state points, because local interfacial pH was not measured. Within this operational scope, the records consistently distinguish the voltage screen and time-series conditions.

### 3.3. Intercolumnar Opening and Narrowing of Vertical Features

**Figure 2:**
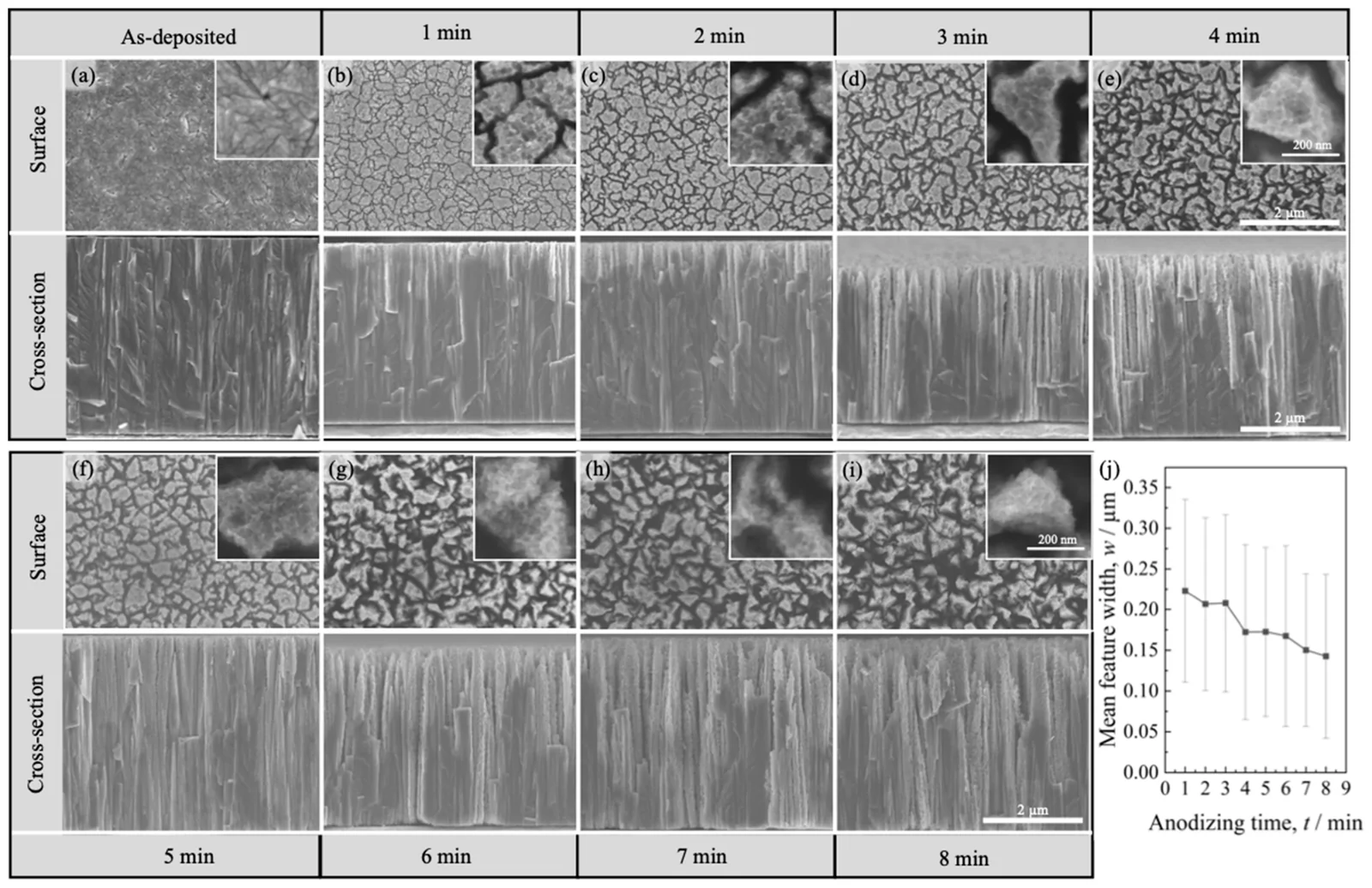
FE-SEM morphology evolution and feature-width quantification during anodization. (**a**–**i**) show the as-deposited coating and the 1–8 min conditions, respectively. For each condition, the upper image is a top view, the inset is the corresponding higher-magnification view, and the lower image is the corresponding cross-section. The submitted 2 µm overview/cross-section and 200 nm inset scale bars are retained. (**j**) shows the within-image mean feature width, with error bars showing the within-image standard deviation. Table S1 reports the mean, median, standard deviation, and coefficient of variation for each condition.

Figure 2 shows the morphological evolution of the as-deposited coating during anodization from 1 to 8 min. Narrow paths initially opened between the closely packed features. With increasing anodization time, these paths broadened and the retained features became laterally thinner. The apparent film height remained between 3.6 and 4.0 µm, close to the initial coating thickness of 4 µm, although some paths reached the Au-coated support by 4 min. The combination of progressive gap widening, limited apparent height change, and localized breakthrough is consistent with spatially non-uniform removal concentrated within the intercolumnar network rather than uniform recession of the top surface [10,11]. Figure S3 presents paired cross-sectional images from separately prepared specimens anodized for 1, 4, and 8 min. These specimens likewise retain the vertical Cu_2_O framework and show greater intercolumnar opening with increasing anodization time. The archival sets were used as qualitative recurrence evidence and remained separate from the quantitative width analysis. Together with the paired surface and cross-sectional observations in Figure S1, Figure S3 shows that characteristic opening recurred across independently prepared specimens and the investigated process window.

The top-view measurements in Figure 2j quantify this lateral narrowing. From 1 to 8 min, the mean feature width decreased from 0.223 to 0.143 µm and the median decreased from 0.202 to 0.109 µm. The decrease in both measures shows that the width distribution shifted toward narrower features rather than being driven only by a few unusually large measurements. Over the same interval, the standard deviation changed from 0.112 to 0.101 µm, while the coefficient of variation increased from 0.503 to 0.705. Absolute dispersion therefore changed only modestly, but the remaining features became more heterogeneous relative to their decreasing mean width. These descriptive statistics were calculated from *n* = 400 image features per condition. Because the measurements describe image features rather than replicate specimens, they were not used to estimate between-specimen variability or inferential uncertainty. Within this defined scope, they provide a consistent quantitative measure of the progressive lateral narrowing shown in Figure 2.

### 3.4. Retained Out-of-Plane Diffraction Signature

**Figure 3:**
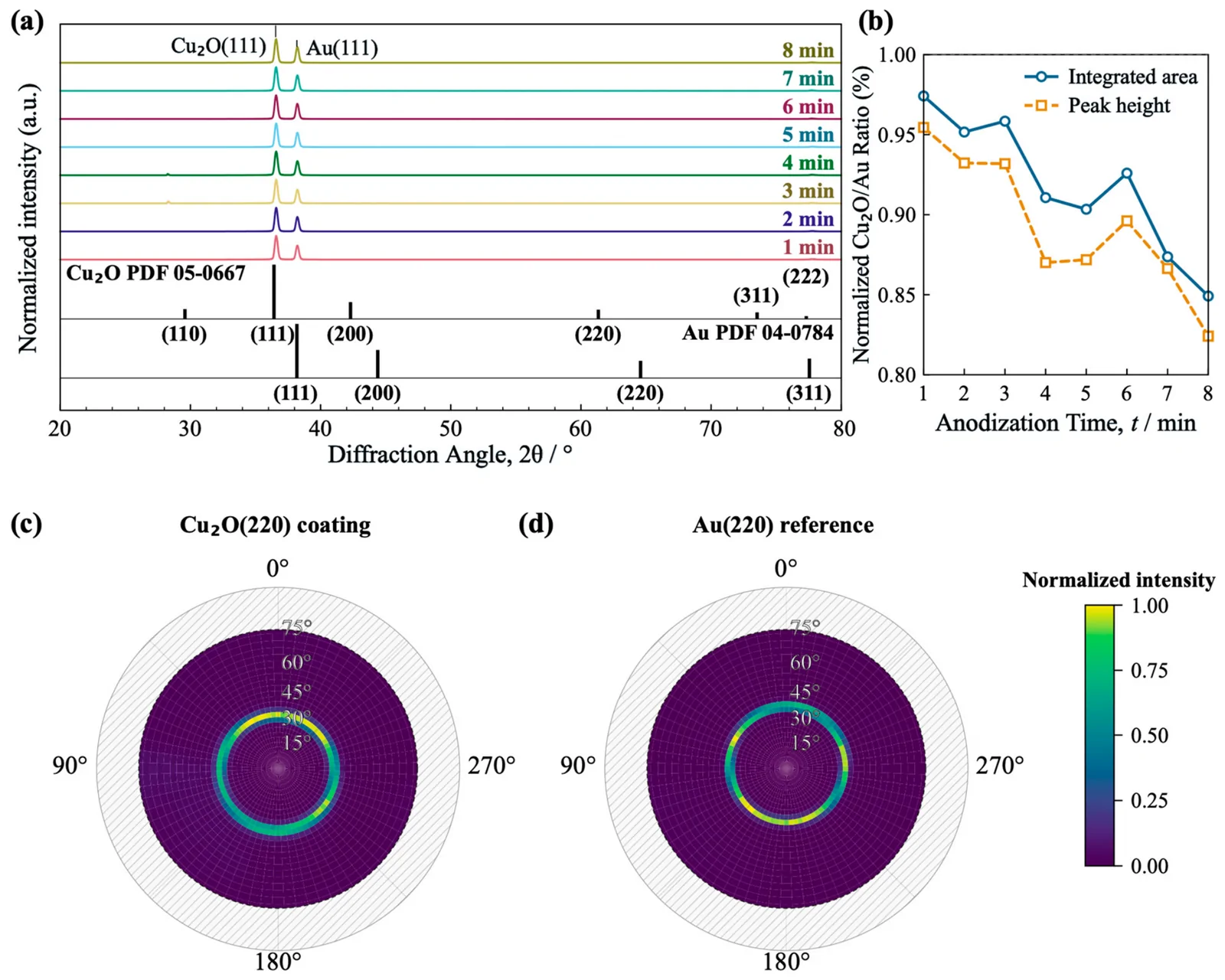
Out-of-plane diffraction ratio retention and pole figure texture context after anodization. (**a**) Anodized out-of-plane θ–2θ XRD profiles for each 1–8 min condition at nominal 10 V over 20–80° 2θ, normalized within each condition and vertically offset. (**b**) Anodized/as-deposited Cu_2_O(111)/Au(111) retention calculated from local baseline integrated areas and the peak height metric. (**c**,**d**) Fixed-2θ (220) pole figures of the Cu_2_O/Au coating at 2θ = 61.3° and the Au reference at 2θ = 64.6°, respectively.

Figure 3a presents the out-of-plane θ–2θ XRD profiles, while Figure 3b shows diffraction ratio retention. Figure 3c,d show the Cu_2_O/Au-coating and Au-reference (220) pole figures. Reference sticks for Cu_2_O (ICDD PDF 00-005-0667) and Au (ICDD PDF 00-004-0784) are shown in separate black lanes at the bottom [14]. Stick heights represent the relative intensity values used in the plotted reference tables, and the hkl labels indicate the reference peak positions. Across the 1–8 min anodization conditions, the profiles retained the Cu_2_O(111) and Au(111) reflections reported for the starting coating. Local edge-linear baselines were applied, and areas were integrated over 35.8–37.3° for Cu_2_O(111) and 37.5–39.1° for Au(111). The stacked profiles were normalized for display. For each condition, Cu_2_O(111)/Au(111) retention is defined by Equation (1) as the post-anodization ratio divided by the corresponding as-deposited ratio. The same definition was applied separately using *A* for integrated peak area and *H* for peak height.

(1) $$R_{A}\left(t\right)=\frac{\frac{A_{Cu_{2}O\left(111\right)}\left(t\right)}{A_{Au\left(111\right)}\left(t\right)}}{\frac{A_{Cu_{2}O\left(111\right)}\left(0\right)}{A_{Au\left(111\right)}\left(0\right)}},\;R_{H}\left(t\right)=\frac{\frac{H_{Cu_{2}O\left(111\right)}\left(t\right)}{H_{Au\left(111\right)}\left(t\right)}}{\frac{H_{Cu_{2}O\left(111\right)}\left(0\right)}{H_{Au\left(111\right)}\left(0\right)}}$$

Equation (1) defines a dimensionless retention ratio in which a value of 1 represents preservation of the starting Cu_2_O(111)-to-Au(111) integrated-area ratio. The integrated area ratio decreased from 0.974 after 1 min to 0.849 after 8 min, while the peak height ratio decreased from 0.954 to 0.824 over the same interval. Integrated area is comparatively tolerant of modest changes in peak width, whereas peak height is more sensitive to peak broadening and changes in peak shape. The agreement between the two metrics indicates that the decrease cannot be attributed solely to peak reshaping. These normalized ratios track the Cu_2_O(111) diffraction contribution relative to the Au(111) reference and the as-deposited coating. The continued presence of the Cu_2_O(111) reflection throughout the 1 to 8 min series shows that the coating’s principal out-of-plane orientation was retained. Together with the FE-SEM observations, these results show that anodization progressively removed material from the columnar coating while the retained features preserved the dominant Cu_2_O(111) diffraction signature.

In the fixed-2θ (220) pole measurements, the Cu_2_O coating and Au reference exhibited annular maxima near ψ = 36°. This agrees with the 35.26° angle between [111] and [220] obtained from the standard cubic lattice scalar product relation in Equation (2) [15], supporting a common [111] film normal. The annular intensity reflects broadly distributed azimuthal orientations about that axis.

(2) $$∠\left(\left[111\right],\left[220\right]\right)=\cos^{-1}\left(\frac{\left[111\right]\cdot \left[220\right]}{\left|\left[111\right]\right|\left|\left[220\right]\right|}\right)=\cos^{-1}\left(\frac{1\cdot 2+1\cdot 2+1\cdot 0}{\sqrt{1^{2}+1^{2}+1^{2}}\sqrt{2^{2}+2^{2}+0^{2}}}\right)=35.264{}^{\circ}$$

A corresponding texture was reported by Zamzuri et al., who evaluated Cu_2_O(111) and Au(111) pole figures and interpreted the azimuthally distributed equivalent {111} poles as evidence of [111] out-of-plane orientation with random in-plane orientation [16]. They also estimated a nominal Cu_2_O(111)/Au(111) lattice mismatch to be 4.7%. In the present coating, the preferred [111] film normal texture, together with the distributed azimuthal orientations, indicates a fiber-textured polycrystalline framework. Within this framework, the vertically aligned columns are separated by an intercolumnar network that provides a structural basis for its preferential widening during anodization.

### 3.5. Optical Response and Secondary Photoelectrochemical Evaluation

**Figure 4:**
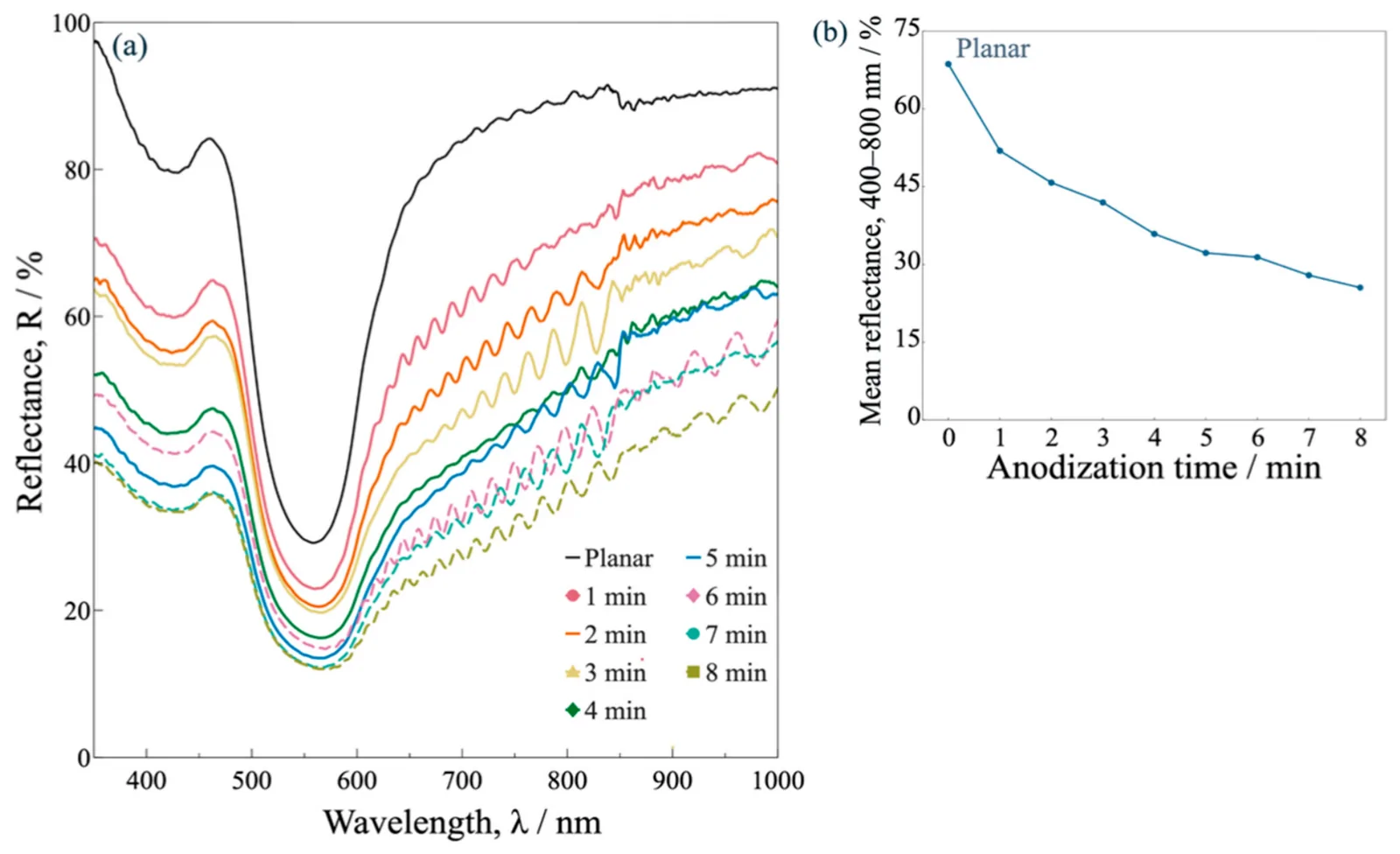
Reflectance response of the planar Cu_2_O coating and anodized coatings. (**a**) Reflectance spectra of the planar Cu_2_O coating and coatings anodized for 1–8 min at 10 V. (**b**) Mean reflectance over 400–800 nm for the same conditions, used for the condition-level comparison. Each FE-SEM, XRD, and UV-visible condition was obtained from a separate planar Cu_2_O coating anodized once for the assigned duration rather than by repeatedly anodizing the same specimen. The separately prepared condition series converge on a consistent duration-dependent trend, which shows greater penetration of intercolumnar opening and substrate exposure, lower Cu_2_O diffraction retention, and lower total reflectance with correspondingly higher apparent absorbance. Together, these observations establish progressive material removal and coupled optical change as anodization time increased.

Figure 4a shows reflectance spectra for the planar coating and every 1–8 min condition, and Figure 4b shows the corresponding arithmetic means over the common 400–800 nm broadband interval. This interval spans the principal visible response and the Cu_2_O absorption-edge region while avoiding the instrument-range endpoints. The planar coating exhibited the expected Cu_2_O absorption-edge region near 650 nm. Mean reflectance decreased from 68.6% for the planar coating to 51.9%, 35.9%, and 25.5% after 1, 4, and 8 min, respectively. Feature separation, roughening, Cu_2_O loss, and increasing substrate exposure occurred concurrently, so the decrease cannot be assigned to a single optical mechanism. The result is reported operationally as the total reflectance collected by the integrating sphere rather than as a measure of scattering, absorption, or substrate exposure alone. The overall decrease in total reflectance establishes that anodization produced a measurable transition to a less reflective Cu_2_O surface.

Following the morphological, structural, and optical comparisons, PEC measurements were used as a secondary functional comparison of the completed coatings. The pH 13.8 application-oriented environment described in Section 2.3 was applied to the planar and all anodized coatings to determine whether microstructuring was accompanied by a measurable change in electrode-level photoresponse. This design clearly distinguishes microstructure formation near pH 6 from the subsequent evaluation of coating function under standardized PEC conditions.

**Figure 5:**
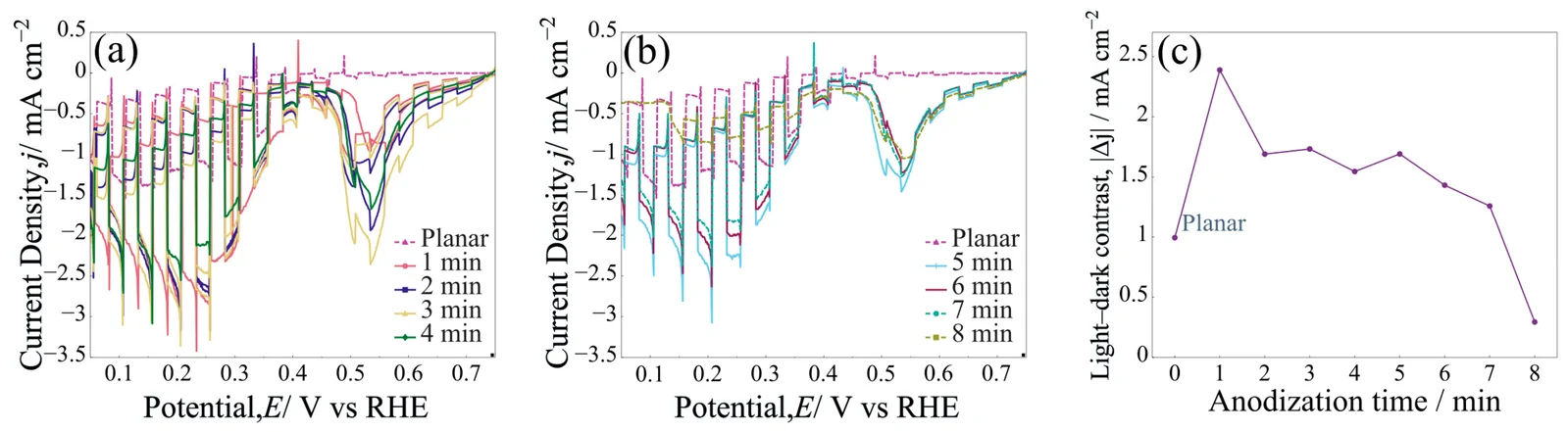
Chopped photoelectrochemical (PEC) response in the pH 13.8 application-oriented electrolyte under AM1.5G illumination. (**a**) Planar and 1–4 min anodization conditions. (**b**) Planar and 5–8 min anodization conditions. (**c**) Condition-series light–dark current density contrast, |Δj|, evaluated near 0.2 V vs. RHE. The common measurement environment enables condition-level functional comparison of the completed coatings after anodization.

Figure 5 shows the chopped PEC responses for the planar coating and the 1–8 min conditions under AM1.5G illumination. The highest illuminated photocathodic current-density magnitude in the present series was 2.849 mA cm^−2^ for the 3 min coating near 0.20 V vs. RHE. For literature context, the optimized Cu_2_O/CuO photocathode protocol cited in Section 2.3 reported 3.15 mA cm^−2^ at 0.40 V vs. RHE. This literature value defines a performance scale, whereas the within-series effect of anodization is evaluated by the light–dark current density contrast, |Δj|, near 0.20 V vs. RHE under identical PEC conditions. |Δj| increased from 0.993 mA cm^−2^ for the planar coating to 2.388 mA cm^−2^ after 1 min. The contrast remained above the planar value for the 2–7 min conditions before decreasing to 0.294 mA cm^−2^ after 8 min. The largest condition-level contrast therefore occurred during early intercolumnar opening, while most of the substrate-connected vertical Cu_2_O framework remained. Increased sidewall access and the subsequent loss or localized breakthrough of Cu_2_O are plausible contributors to this non-monotonic association. Current density normalized by exposed geometric area provides a consistent electrode-level comparison under the common PEC conditions. Electrochemically active area, carrier transport and recombination, and specimen-specific electrical connectivity were not measured, so their individual contributions are not separated. These records show that the completed coatings retained measurable photoresponse and that the largest condition-level contrast occurred after short anodization.

A cathodic feature near 0.55 V vs. RHE was observed for the anodized coatings but not for the planar coating. Its appearance after anodization, together with the XPS Cu(II)/CuO-like contribution presented in Section 3.6, supports its assignment to reduction of an anodization-associated Cu(II)-rich surface contribution toward Cu(I).

### 3.6. Evidence-Bounded Interpretation of Intercolumnar Material Removal

FE-SEM directly shows progressive widening of the pre-existing intercolumnar network. At the coating morphology scale, this spatial evolution identifies the network as the principal removal pathway and supports preferential dissolution along that network [10,11]. The individual paths were not crystallographically resolved. Declining Cu_2_O diffraction retention and increasing charge provide complementary evidence of continued material removal. Together, the morphology and bulk records establish progressive removal through the intercolumnar network while the vertical framework is retained over the useful anodization window.

**Figure 6:**
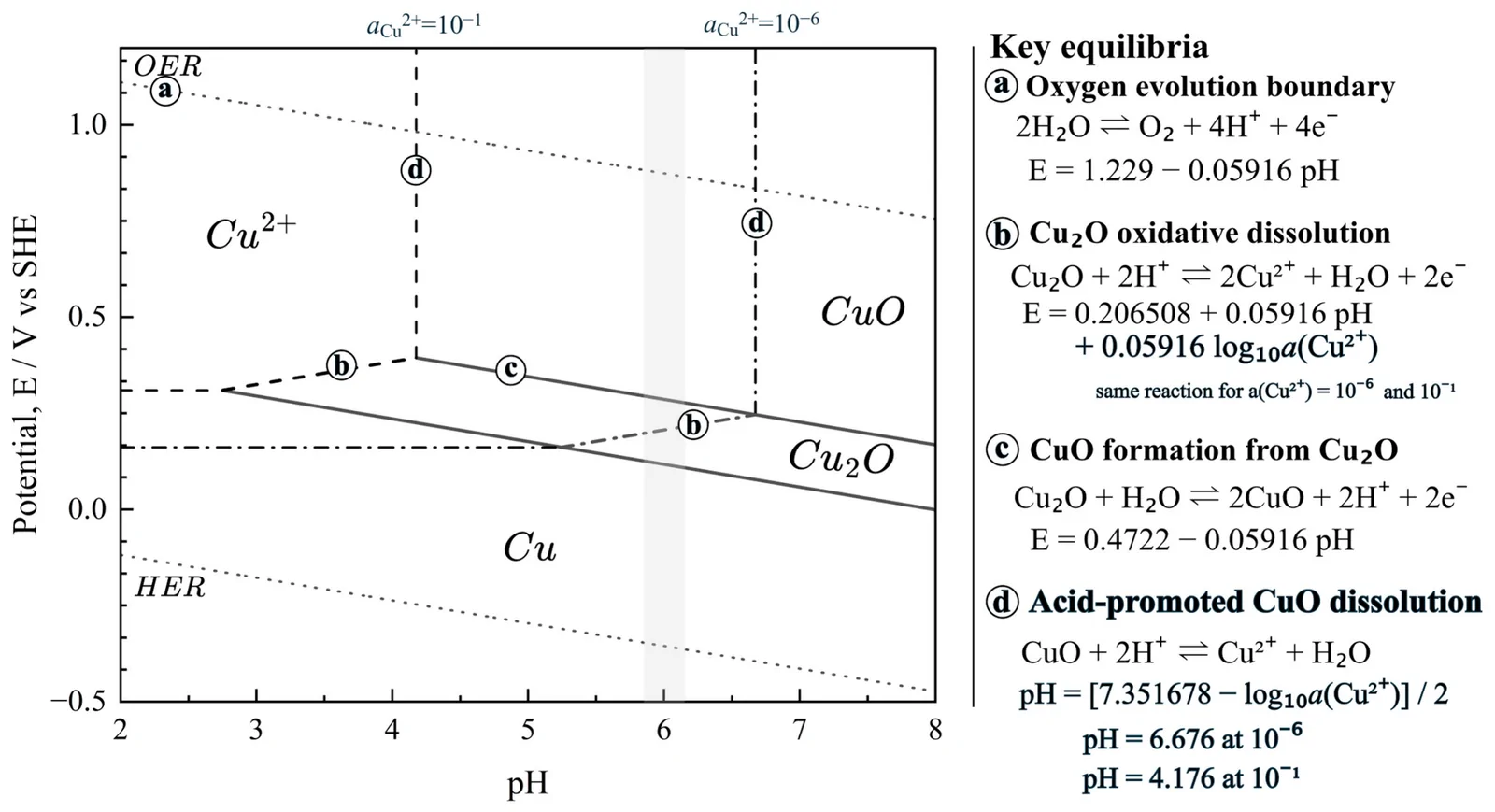
Cu-H_2_O potential pH framework at 298 K for Cu^2+^ activities of 10^−6^ and 10^−1^. The shaded region marks the recorded bulk electrolyte pH near 6, and boundaries (a–d) correspond to Equations (3)–(6).

Figure 6 presents the Cu-H_2_O potential-pH framework relevant to anodization [17,18]. The shaded region marks the recorded bulk electrolyte pH near 6, while the working electrode readings include uncompensated resistance. It should be noted that the true interfacial potential cannot be determined from those readings without iR correction, and local pH was not measured. Figure 6 therefore serves as a qualitative thermodynamic guide to possible oxidative transformations rather than a quantitative description of the instantaneous interfacial state. Within this defined role, it connects the recorded bulk pH, applied anodic direction, and observed oxygen evolution to the experimentally supported routes for oxidative material removal.

Figure 6 labels four relevant equilibria as (a)–(d). They correspond to the oxygen evolution boundary, oxidative dissolution of Cu_2_O to soluble Cu(II), oxidation of Cu_2_O to CuO, and acid-promoted dissolution of CuO, respectively. Equations (3)–(6) present the balanced reactions using the same cited thermodynamic basis [17,18]:

(3) $$2H_{2}O⇌O_{2}+4H^{+}+4e^{-}$$

(4) $${Cu}_{2}O+2H^{+}⇌2{Cu}^{2+}+H_{2}O+2e^{-}$$

(5) $${Cu}_{2}O+H_{2}O⇌2CuO+2H^{+}+2e^{-}$$

(6) $$CuO+2H^{+}⇌{Cu}^{2+}+H_{2}O$$

**Figure 7:**
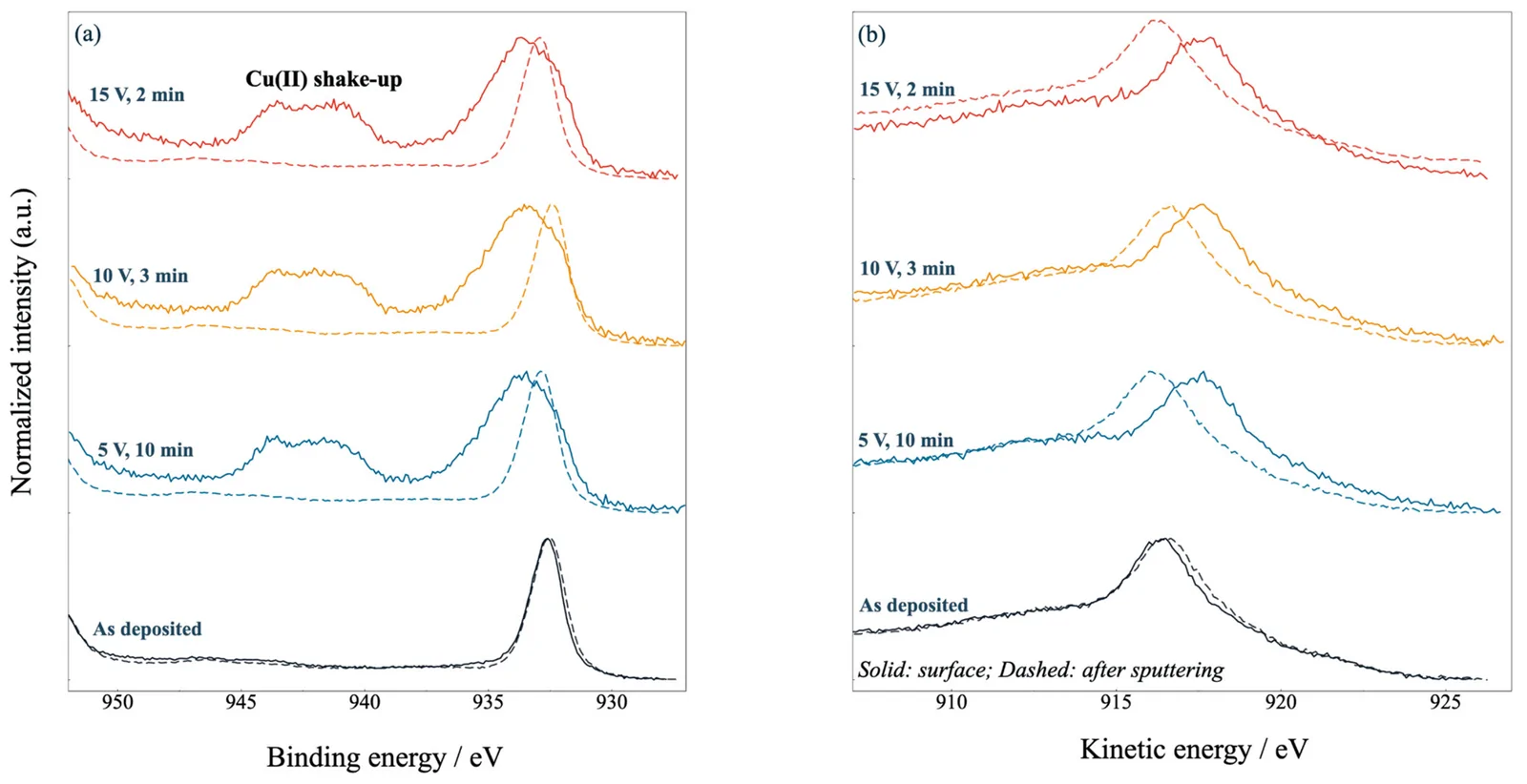
C 1s-referenced XPS spectra (C 1s = 284.8 eV) for the as-deposited coating and specimens anodized at nominal 5 V for 10 min, 10 V for 3 min, and 15 V for 2 min. (**a**) Cu 2p_3/2_ spectra at the as-received surface (solid) and after Ar^+^ sputtering (dashed). (**b**) Corresponding Cu LMM spectra.

Vigorous microbubble formation during anodization supports operation in the oxygen-evolving regime represented by equilibrium (a). Equilibria (b)–(d) define two accessible material removal routes. One involves direct oxidation of Cu_2_O to dissolved Cu(II). The other proceeds through a CuO-like surface state followed by dissolution. The blue electrolyte clouding is consistent with copper transfer from the solid coating into the electrolyte [19,20]. The framework also aligns with the progressive gap widening and feature narrowing observed by FE-SEM. Together, these observations provide a coherent explanation for oxidative material removal expressed preferentially through the intercolumnar network. Figure 7 provides ex situ XPS evidence for the CuO-like surface state represented in this framework.

Figure 7 shows that Cu(II)-type shake-up intensity was absent or weak at the as-deposited surface. It appeared at every anodized endpoint and was strongest at the 15 V endpoint despite the shorter anodization time. Binding energy and Auger peak shifts were followed by strong attenuation after sputtering. This behavior is consistent with an anodization-associated Cu(II)/CuO-like contribution concentrated at the outermost surface [21,22]. The voltage screen trend provides direct ex situ support for the CuO-forming branch in Figure 6 and for surface oxidation driven by stronger polarization.

Reports of chemically distinct Cu_2_O boundary regions and orientation-dependent etching provide plausible context for spatially non-uniform attack [10,11]. Particle-scale studies also show environment- and plane-dependent Cu_2_O dissolution [23,24,25]. These independent reports establish that spatially non-uniform dissolution can occur in Cu_2_O under other material and electrolyte conditions. In the present study, the interpretation is grounded directly in the coating morphology, where progressive opening of the intercolumnar network expresses preferential dissolution at the coating scale.

Charge-derived Faradaic upper bounds and the cited Faraday’s law basis are provided in the Supporting Information. Oxygen evolution shares the anodic current. The values therefore define the maximum theoretical removal scale allowed by the charge record, not a measured mass loss, dissolved copper quantity, or dissolution efficiency. In this role, they provide a useful quantitative consistency check against the observed morphology evolution.

Anodization time is therefore used as the primary descriptor for the transition from a dense columnar coating to separated vertical features and, after prolonged anodization, structural deterioration. Charge is a complementary process record. Microscopy provides the direct spatial evidence, while XRD, XPS, and the Cu-H_2_O framework together connect the structural and surface changes to thermodynamically accessible oxidative removal pathways.

### 3.7. Scope and Outlook

Prior studies have established acidic etching of electrodeposited Cu_2_O photocathodes and Cu^+^ dissolution during anodic Cu_2_O growth on Cu [26,27]. Orientation-dependent ammonia etching of thermally oxidized Cu_2_O has also been demonstrated [10]. The present study differs by applying oxygen-evolving anodization after deposition to open an existing columnar network. This Cu_2_O result establishes a two-step route in which electrodeposition forms a columnar framework and subsequent anodization opens its pre-existing intercolumnar network. This process architecture provides a basis for exploring analogous post-deposition structuring strategies in other electrodeposited crystalline-oxide coatings.

Building on the demonstrated control of columnar feature width, spacing, sidewall exposure, and roughness, future oxide-specific studies may test wetting [28], Cu^+^-mediated antimicrobial interfaces [29], or geometry-dependent mechanobactericidal contact after refinement to the relevant length scale [30].

## 4. Conclusions

A two-step, all-solution process converted an electrodeposited columnar Cu_2_O coating with a thickness near 4 µm into substrate-supported vertical microfeatures. Anodization in dilute peroxydisulfate progressively opened the pre-existing intercolumnar network. FE-SEM showed wider gaps and narrower retained features, establishing the intercolumnar network as the principal removal pathway at the coating morphology scale and supporting preferential dissolution along the intercolumnar network. From 1 to 8 min, mean feature width decreased from 0.223 to 0.143 µm and median width from 0.202 to 0.109 µm. The normalized Cu_2_O(111)/Au(111) integrated area ratio decreased from 0.974 to 0.849, showing progressive loss of diffracting Cu_2_O while the dominant out-of-plane Cu_2_O(111) signature persisted.

Chopped PEC measurements provided a secondary post-anodization functional comparison. The largest condition-level light–dark contrast occurred after 1 min and remained above the planar value through 7 min, showing that completed coatings retained measurable photoresponse across most of the sequence. C 1s-referenced XPS showed an anodization-associated, surface-enriched Cu(II)/CuO-like contribution that diminished after Ar^+^ sputtering. Together, FE-SEM morphology, XRD retention, XPS surface chemistry, and the Cu-H_2_O framework support high-potential oxidative removal that opens the intercolumnar network while retaining the vertical framework during early and intermediate anodization.

The central contribution is a parallel, post-deposition electrochemical microstructuring route for a columnar Cu_2_O coating. Because anodization acts across the exposed electrode area, the process avoids serial feature writing while retaining a substrate-supported vertical framework during early and intermediate anodization.

## Figures and Tables

**Table 1:** Principal deposition and electrochemical microstructuring conditions used in this study.

Electrolyte	Electrical Condition	Temperature	Stage
0.4 M Cu acetate + 3.0 M lactic acid, pH 12.5	−0.4 V vs. Ag/AgCl to −3.0 C cm^−2^	313 K	Cu_2_O deposition
0.002 M Na_2_S_2_O_8_, pH 6	5, 10, and 15 V (equipment setting) 10, 3, and 2 min respectively	277 K	Voltage screen
0.002 M Na_2_S_2_O_8_, pH 6	10 V (equipment setting) 1–8 min	277 K	Time series

## Data Availability

The data supporting the findings of this study are contained within the article and its Supplementary Materials. Source workbooks and available measurement records are available from the corresponding author upon reasonable request.

## Supplementary Materials

The following supporting information can be downloaded at https://www.mdpi.com/article/10.3390/mi17080979/s1, The Supplementary Materials contain Figure S1, which presents the operational voltage screen curves, photographs, recorded charge, and paired surface and cross-sectional FE-SEM images at the 5, 10, and 15 V endpoints. Figure S2 presents the 1–8 min current density and working electrode records. Figure S3 shows qualitative recurrence of cross-sectional morphology in independently prepared specimens anodized for 1, 4, and 8 min. Table S1 reports within-image feature-width statistics. Table S2 reports representative full-series PEC values. Reference [13] is cited in the Supplementary Materials.
