# Supplementary material for: Electrochemical Microstructuring of Columnar Cu2O Layers Through Preferential Grain Boundary Dissolution
Source: Micromachines (Basel). 2026 Aug 19;17(8):979. doi: 10.3390/mi17080979 (PMC13515733; doi:10.3390/mi17080979)
Supplement: Supplementary file 1 [file micromachines-17-00979-s001.zip › micromachines-4505818-supplementary.pdf]

Supporting Information

Electrochemical Microstructuring of Columnar Cu_2O Layers through Preferential Grain-Boundary Dissolution

Pei Loon Khoo, Mizuki Kono, Katsutoshi Sakai, Masakazu Kobayashi, and Masanobu Izaki

Figure S1

The current-density responses in Figure S1(a) increased rapidly after application of the nominal cell voltage and subsequently approached condition-dependent quasi-steady values. Increasing the voltage from 5 to 15 V produced a higher anodic current density, while the anodization duration was shortened to limit excessive material removal. The corresponding charge densities were 1.842, 1.601, and 2.081 C cm^{-2} for the 5 V–10 min, 10 V–3 min, and 15 V–2 min conditions, respectively. The endpoint photographs in Figures S1(b)–(e) show visible changes in the coating appearance after anodization. Figures S1(f)–(h) add paired surface and cross-sectional FE-SEM images from independently prepared coatings at the same three voltage-screen endpoints. For consistent visual comparison, the same 1040 × 820 pixel central crop was applied to all six source fields, and each 1 μm scale bar was calibrated from the original 2.00 μm instrument ruler retained in the corresponding source file and generated by proportional scaling. At every endpoint, the surface view shows the opened intercolumnar network and the cross-section shows retention of the vertical Cu_2O framework. Together with Figure S3, these observations provide qualitative evidence that intercolumnar opening recurred across independently prepared specimens and the investigated process window, while the two archival sets remain separate from matched quantitative replication. The current-density traces are interpreted as total anodic demand, which includes oxygen evolution and other competing processes, and the paired microscopy provides the spatial evidence of Cu_2O removal.

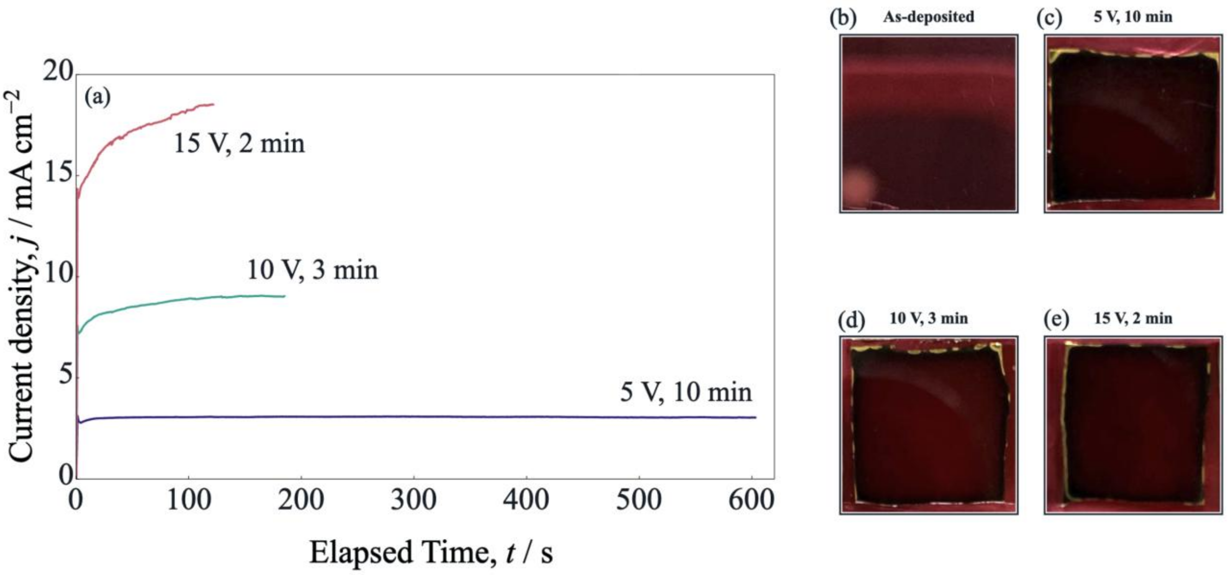

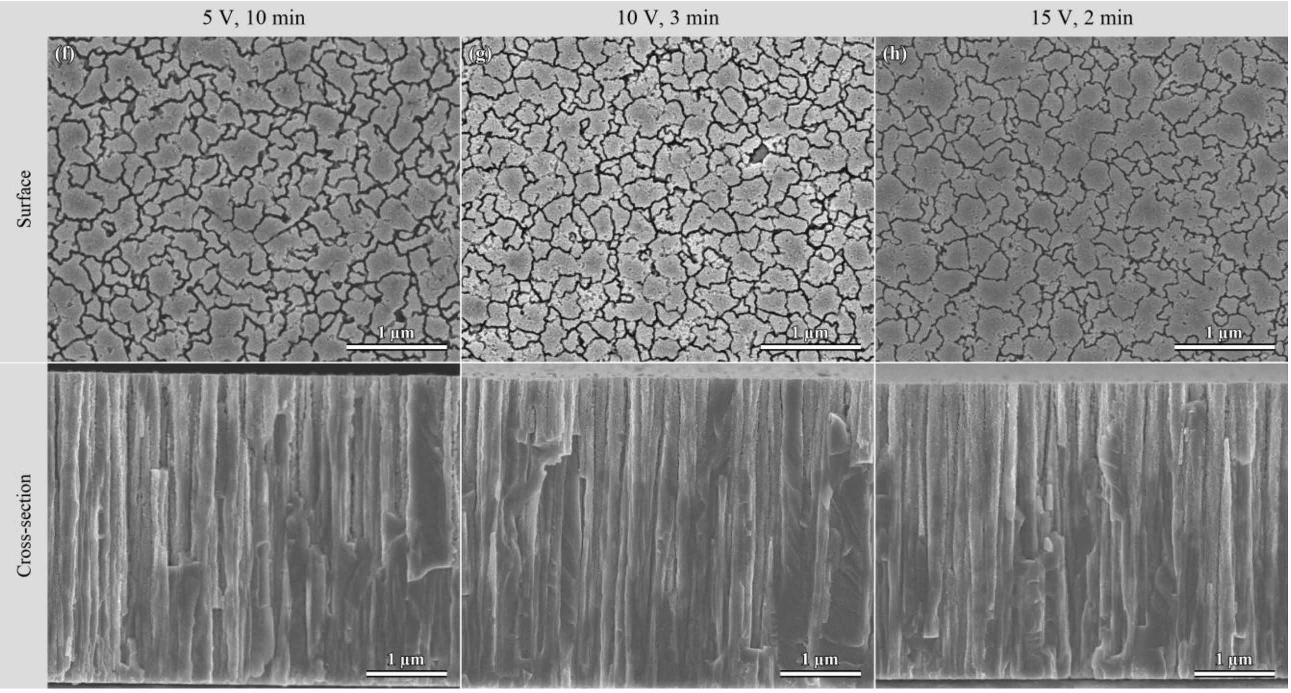

Figure S1. Operational voltage screen and corresponding specimen appearance and morphology. (a) Current-density traces for nominal 5 V (10 min), 10 V (3 min), and 15 V (2 min). (b)–(e) Photographs of the as-deposited coating and the 5, 10, and 15 V endpoint specimens, respectively. (f)–(h) Paired surface and cross-sectional FE-SEM views of independently prepared coatings at the same endpoints. Scale bars: 1 µm.

Figure S2

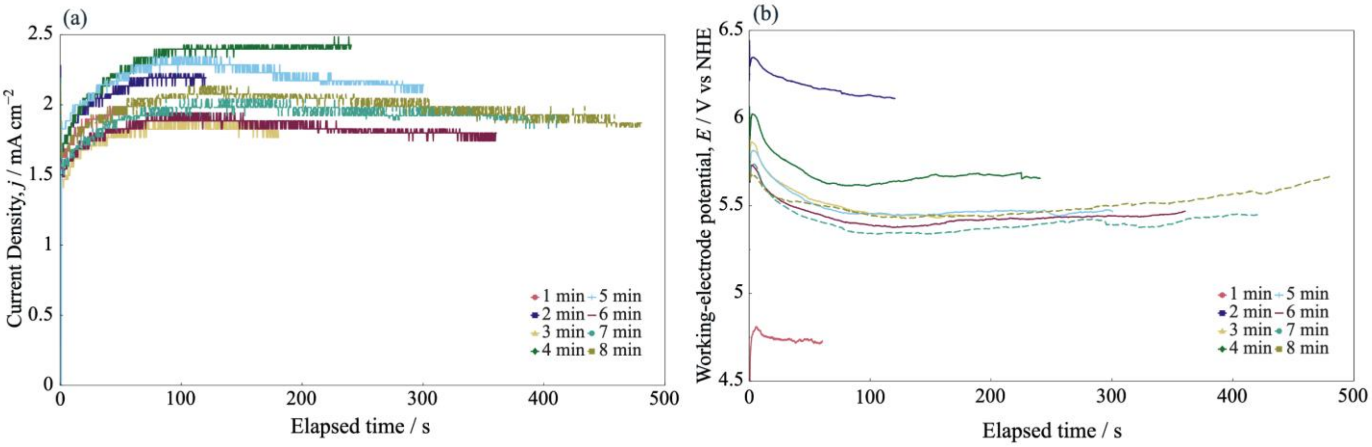

Figure S2. Electrochemical records for the 1–8 min anodization series at nominal 10 V in 0.002 mol L^{-1} $Na_2S_2O_8$ at 277 K. (a) Current-density–time curves. (b) Working-electrode-potential–time curves versus NHE.

Figure S2(b) records a rapid increase in the working-electrode reading followed by condition-dependent quasi-steady values, providing an operational description of gas-evolving anodization. The readings include uncompensated resistance and gas coverage, and local interfacial pH was not measured. They are therefore interpreted as instrument-level process records. Within this role, the potential and current traces consistently distinguish the gas-evolving anodization conditions.

The cumulative anodic charge density, q_A, was obtained by integrating the current-density records in Figure S2(a) over anodization time. A maximum theoretical Cu_2O-removal scale was then estimated from Faraday's law of electrolysis [13]. The charge integration is written as:

$$q_\mathrm{A} = \int_0^{t_\mathrm{f}} j(t)\ \mathrm{d}t$$

Equation S1

where q_A is the cumulative anodic charge density, *j(t)* is the measured current density, and t_f is the anodization duration. The resulting charge densities were 0.113 C cm^{-2} after 1 min and 0.951 C cm^{-2} after 8 min. Assigning all anodic charge to two-electron Cu_2O oxidation ($n = 2$) gives the following upper-bound Cu_2O mass loss per unit area:

$$m_{\mathrm{Cu_2O}} = \frac{q_\mathrm{A} M_{\mathrm{Cu_2O}}}{nF}$$

Equation S2

where $M_{\mathrm{Cu_2O}}$ = 143.09 g mol^{-1} is the molar mass of Cu_2O, n = 2 is the number of electrons per formula unit, and F = 96485 C mol^{-1} is the Faraday constant. Equation S2 gives upper bounds of 0.084 and 0.71 mg Cu_2O cm^{-2}, equivalent to 0.14 and 1.18 µm of dense-planar-film-equivalent removal for a Cu_2O density of 6.0 g cm^{-3}.

Because oxygen evolution and other anodic processes share the measured current, the calculation gives a Faradaic upper bound rather than a measured dissolution yield. This upper-bound scale is compatible with the substantial but incomplete material removal indicated by FE-SEM.

13. Bard, A.J.; Faulkner, L.R.; White, H.S. *Electrochemical Methods: Fundamentals and Applications*; Third edition.; Wiley: Hoboken, NJ, USA Chichester, West Sussex, UK, 2022; ISBN 978-1-119-33406-4.

Table S1

Table S1 summarizes 400 feature-width measurements for each anodization duration. The mean represents the average measured width, whereas the median represents the typical width and is less sensitive to unusually large values. The standard deviation describes absolute dispersion, and the coefficient of variation (standard deviation divided by the mean) describes dispersion relative to the mean. From 1 to 8 min, the mean and median decreased, the standard deviation changed from 0.112 to 0.101 μm, and the coefficient of variation increased from 0.503 to 0.705. The concurrent decreases in mean and median indicate a within-image shift toward narrower features, while the increasing coefficient of variation indicates greater relative heterogeneity. Because each condition represents 400 image features rather than independent specimens, these statistics describe within-image distributions. Within that scope, they quantify the duration-dependent narrowing and increasing relative heterogeneity.

Table S1. Descriptive feature-width statistics for anodization times of 1–8 min.

Condition	n	Mean (μm)	Median (μm)	SD (μm)	CV
1 min	400	0.223	0.202	0.112	0.503
2 min	400	0.207	0.191	0.106	0.514
3 min	400	0.208	0.185	0.109	0.524
4 min	400	0.172	0.145	0.107	0.624
5 min	400	0.173	0.151	0.104	0.601
6 min	400	0.168	0.140	0.111	0.663
7 min	400	0.150	0.129	0.094	0.624
8 min	400	0.143	0.109	0.101	0.705

Table S2

Table S2 summarizes the originally presented condition-series values near 0.2 V vs. RHE. All coatings were evaluated in the same alkaline PEC electrolyte, providing a standardized post-anodization functional comparison, while the morphology and pH near 6 process records remain the direct basis for interpreting anodization.

Table S2. Representative photoelectrochemical values near 0.2 V vs. RHE.

Condition	Illuminated current (mA cm^{-2})	Dark current (mA cm^{-2})	Light–dark difference (mA cm^{-2})
Planar	-1.188	-0.195	0.993
1 min	-2.800	-0.412	2.388
2 min	-2.660	-0.968	1.691
3 min	-2.849	-1.116	1.733
4 min	-2.293	-0.746	1.547
5 min	-2.399	-0.707	1.692
6 min	-2.105	-0.672	1.433
7 min	-1.931	-0.671	1.260
8 min	-0.846	-0.552	0.294

Figure S3

Figure S3 compares paired cross-sectional FE-SEM fields from separately prepared specimens anodized for 1, 4, and 8 min. For consistent visual comparison, the same 1040 × 820 pixel central crop was applied to all six source fields, and each 1 μm scale bar was calibrated from the original 2.00 μm instrument ruler retained in the corresponding source file and generated by proportional scaling. The vertical framework remains visible and intercolumnar opening increases with anodization time. Together with Figure S1, the paired fields provide qualitative recurrence evidence across independently prepared specimens and the investigated process window while remaining separate from the 400-feature width analysis.

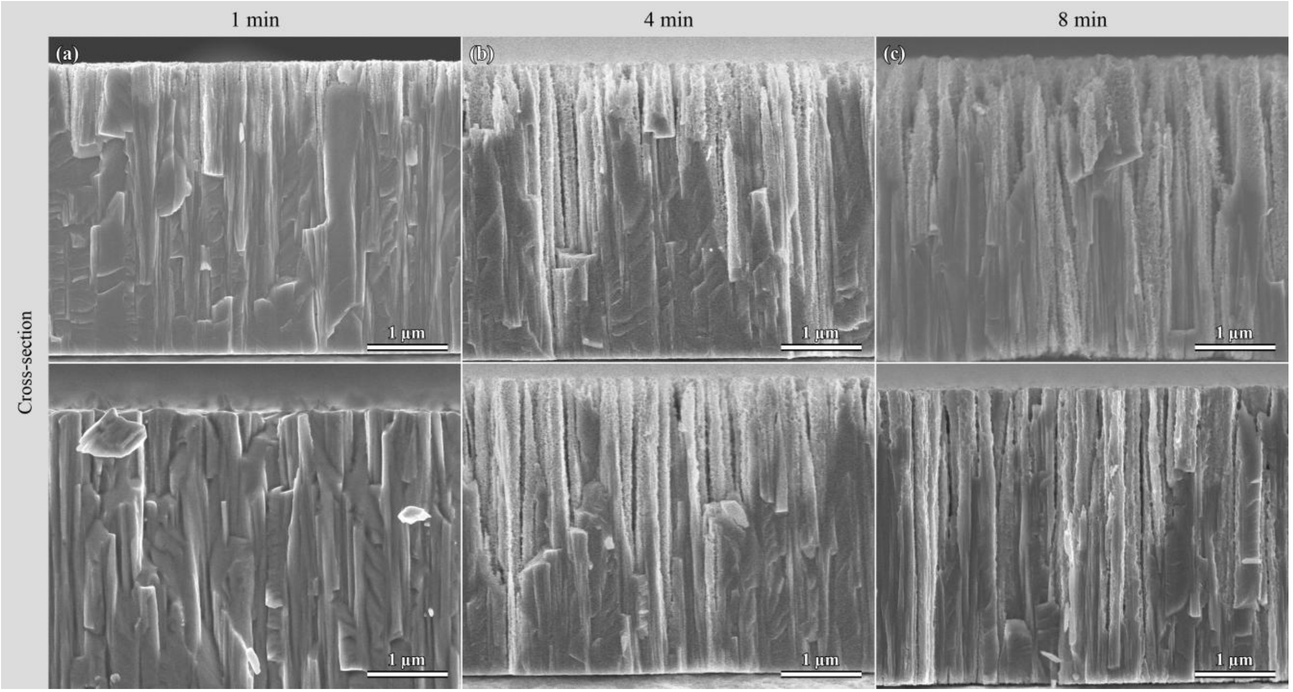

Figure S3. Cross-sectional FE-SEM morphology of independently prepared specimens anodized for 1, 4, and 8 min. Figures S3(a)–(c) show paired fields for each duration. Scale bars: 1 μm. Anodization conditions: 0.002 mol L^{-1} $Na_2S_2O_8$, nominal 10 V, and 4 °C.
